# Molecular Epidemiology of Pathogenic *Leptospira* spp. Infecting Dogs in Latin America

**DOI:** 10.3390/ani13152422

**Published:** 2023-07-27

**Authors:** Maria Isabel Nogueira Di Azevedo, Luiza Aymée, Ana Luiza dos Santos Baptista Borges, Walter Lilenbaum

**Affiliations:** Laboratory of Veterinary Bacteriology, Biomedical Institute, Federal Fluminense University, Niterói, Rio de Janeiro 24020-150, Brazil; isabeldiazevedo@gmail.com (M.I.N.D.A.); luizaaymeeps@gmail.com (L.A.); analuizaborges@id.uff.br (A.L.d.S.B.B.)

**Keywords:** canine leptospirosis, bioinformatics, *sec*Y, 16S rRNA, one health

## Abstract

**Simple Summary:**

Leptospirosis is a neglected zoonosis caused by a bacterium of the genus *Leptospira.* Dogs are highly susceptible to infection, which can range from chronic and asymptomatic to acute and severe disease. Although serosurveys of canine leptospirosis in Latin America are widely published, the gathering of molecular data of strains identified from dogs has not yet been performed. Based on *Leptospira* spp. DNA sequences collected on digital platforms, we aimed to genetically analyze the strains circulating in Latin America in order to provide an unprecedented overview of the molecular epidemiology of pathogenic leptospires infecting dogs in the region. We found a very high genetic similarity between strains apart serogroup, clinical signs, or geographical localization. The strains were similar to the one that most circulates in humans. In addition to the importance from a zoonotic point of view, this high genetic similarity between strains can, in theory, facilitate the development of an effective broad-spectrum vaccine across the continent, as well as enable the standardization of rapid diagnostic tests.

**Abstract:**

Canine leptospirosis is a bacterial disease caused by spirochetes of the genus *Leptospira*. Infections can vary from asymptomatic and chronic infections to clinical acute diseases. The disease is endemic in tropical areas, such as Latin American countries, but a broad understanding of the dynamics of circulation of strains, based on molecular data, has not yet been performed. Based on in silico analyses, the present study aims to analyze the genetic diversity and circulation patterns of haplotypes from pathogenic leptospires infecting dogs in Latin America. DNA sequences were obtained from GenBank platform, curated, and aligned. Genetic distances were calculated, and a maximum likelihood tree and haplotype network were constructed. According to the inclusion criteria adopted, a total of 148 sequences were identified. Most of the records were from Brazil, including sequences from *L. interrogans* serogroup Icterohaemorrhagiae. Phylogenetic analysis showed a genetically closely related cluster, consisting of a larger haplogroup that includes the reference strain Fiocruz L1-130, known to be the major circulating strain in humans. Moreover, no genetic variations were observed according to clinical history and/or geographical localization. We described the molecular epidemiology of leptospires circulating among dogs in Latin America and demonstrated a very genetically homogeneous group, elucidating its ubiquitous circulation pattern and drawing attention to the important role of dogs in the One Health transmission dynamics of leptospirosis.

## 1. Introduction

Leptospirosis is a neglected zoonosis caused by bacteria of the genus *Leptospira,* and affects domestic and wild animals, as well as humans [1]. Leptospires have a thin and spiral morphology, presenting 6 to 20 µm length and two periplasmatic flagella, producing their unique motility [2,3]. The presence of lipopolysaccharides in their outer membrane classifies the genus as Gram-negative [2,4]. The leptospires can be classified either by their serological or by genetic features. Serological classification is based on the homogeny of LPS antigen between serovars of a serogroup [5]. Regarding genetic classification, the leptospiral species are currently divided into approximately 68 genomospecies separated in pathogenic (subclade P1), intermediate (subclade P2), and saprophytic (subclades S1 and S2) [6,7]. Based on both molecular and serological characterization, 17 pathogenic species and approximately 300 serovars grouped in 24 serogroups, have been identified [6,8]

The life cycle of leptospires is complex, including the natural environment, asymptomatic reservoirs, and susceptible hosts [9]. Due to the great variety of strains, leptospires can infect a wide range of animal species and lead to a potentially lethal disease [10,11]. Regarding human infection, it infects more than 1 million people and is responsible for about 60,000 deaths per year worldwide [1]. Human leptospirosis has diverse clinical manifestations, ranging from a mild, self-limiting acute febrile illness to a severe, life-threatening condition with multiple organ dysfunction [12]. In urban areas, the transmission is directly associated to the proximity with rodent populations, especially the brown rat (*Rattus norvegicus*), the most important source of human infections [12]. The characteristics of urban poor communities (e.g., open sewers, accumulated trash, and dirt floors) create a habitat ideal for rats, which leads to high infestation rates and frequent contact with residents [13].

Transmission occurs by direct contact with infected animals, or with water or soil contaminated with leptospires (shed in the urine of infected animals) via penetration through cuts or abrasions in the skin and/or external mucous membranes such as oral, conjunctiva, respiratory, and genitourinary [14]. Although leptospires do not replicate outside the host and are easily inactivated under adverse conditions, pathogenic serovars can remain viable in water or soil for weeks [15].

Due to the great variety of strains, leptospires can infect a wide range of animal species and lead to a potentially lethal disease [11,12]. Historically, the etiology of leptospirosis infections is divided into those determined by adapted or incidental strains. Adapted strains are harbored by the usual animal host, with a long process of pathogen-host coevolution, leading to asymptomatic or inapparent disease, or mild and subclinical symptoms of a chronic nature. Maintenance hosts indicate the infected animals which are asymptomatically infected and shed viable leptospires in their fluids, acting simultaneously as hosts and reservoirs [16]. Those hosts play an important role in the epidemiology of leptospirosis, as they harbor and excrete leptospires in the environment for long periods, becoming important sources of infection of leptospires of virulent strains to non-adapted to incidental hosts [17,18]. Examples of adapted serovars are Bratislava in horses, Pomona in pigs, Hardjo in ruminants, and Canicola in dogs.

Dogs have been recognized as hosts of pathogenic leptospires since 1931 when Klarenbeek and Schuffner first isolated the bacterium from the urine of a dog affected by nephritis [19]. Currently, canine leptospirosis has been reported worldwide [18,20]. Dogs are highly susceptible to infection by leptospires, which can be maintained in the kidneys and shed through urine and other body fluids, elucidating its important role as source of infection for human infection [21]. Clinical manifestations in dogs range from chronic and asymptomatic infection to acute and severe disease [10,22]. Renal involvement ranges from acute to chronic kidney injury and can be characterized by polyuria, with or without azotemia, or even oliguria and anuria [18,23]. Regarding the hepatic manifestations, leptospirosis can cause mild enzyme alterations to severe failure, with hepatic encephalopathy and jaundice [24,25]. Fever and pain are associated, and can be caused by myositis, meningitis, or other inflammations [15,18]. Cardiac and respiratory manifestations can also occur, such as pulmonary hemorrhage [26]. The incubation is approximately seven days in experimental studies, varying according to the host immune response, virulence, and inoculum [27,28].

Infection by *L. interrogans* serogroup Icterohaemorrhagiae has been assigned to be the major cause of the acute signs, while adapted strains of *L. interrogans* serogroup Canicola are more frequently associated with mild disease [21,22,29]. Nevertheless, not only the virulence of the strain determines the severity of the infection. Recent evidence demonstrated that the same strain can provoke clinically severe features or induce an asymptomatic infection in different dogs [30]. This study analyzed genetic diversity on the *sec*Y gene of *L. interrogans* serogroup Icterohaemorrhagiae strains obtained from 13 dogs, including animals with signs of acute disease, asymptomatic, and presenting chronic kidney disease. There were no differences between the groups’ sequences.

Despite the clinical outcomes, infected animals shed leptospires in the environment, promoting the exposure of other mammals [10,31]. Due to the close proximity to humans, infected dogs play an important role in One Health [32], especially in the cases of asymptomatic harboring [22]. The real impact of canine leptospiral infection and its transmission to humans is poorly understood [32,33]. Nonetheless, reports show that leptospirosis is endemic in tropical areas, such as Latin American countries [21]. Serosurveys of canine leptospirosis in those countries are widely published. In Nicaragua, an epidemiological study from 2007 to 2013 revealed the influence of serogroups Canicola, Pyrogenes, and Louisiana in canine leptospirosis [34]. A serosurvey in Colombia found Autumnalis and Canicola as the most prevalent serovars [35]. Still, seroprevalence studies in Chile reported the presence of Canicola, Pomona, and Autumnalis in both urban and rural areas [36,37]. Isolates were recovered from urine (*n* = 6), blood (*n* = 1), and fetus (*n* = 1) in Argentina. The genetic profile of the strains was identical to *L. interrogans* serogroup Canicola, *L. interrogans* serogroup Icterohaemorrhagiae and *L. interrogans* serogroup Pomona [38]. Recently, a review conducted in Brazil revealed the serogroups Canicola, Icterohaemorrhagiae, and Autumnalis as the most prevalent [29].

As abovementioned, most of studies regarding leptospiral infection in dogs are based on serodiagnosis tests. Serology constitutes an indirect method, and its epidemiological value resides in its ability to predict the circulating serogroups. Even though useful for a collective diagnosis, it presents important limitations for chronic animal leptospirosis, and is not adequate for an individual diagnostic, as serological and molecular results are very often discrepant, and many infected animals may present seronegative. In this context, the complementary use of molecular tools has been encouraged [39]. In the last decades, DNA sequencing has become less costly and more streamlined by automating processes. In addition, data are being increasingly deposited on free-access digital platforms, making it possible to perform advanced studies through bioinformatics tools. Currently, despite a representative sequence bank available, the gathering of molecular data of strains identified from dogs has not yet been performed. As a consequence, Caimi et al. [34] were able to perform a study involving almost 500 leptospiral genomes from worldwide, applying R7L-MLST and obtaining important insights in the phylogenetic and epidemiological scenarios. Therefore, based on in silico analyses, the present study aims to analyze the genetic diversity and circulation patterns of haplotypes from pathogenic leptospires infecting dogs in Latin America.

## 2. Materials and Methods

### 2.1. Collection of Genetic Data

In January 2023, DNA sequences were obtained from the GenBank platform (NCBI/nucleotide) using the following search terms: “*Leptospira*” AND “dog” OR “*Canis familiaris*” OR “*Canis lupus familiaris*” OR “canine” AND “Argentina” OR “Bolivia” OR “Brazil” OR “Chile” OR “Colombia” OR “Costa Rica” OR “Cuba” OR “Ecuador” OR “El Salvador” OR “Guatemala” OR “Haiti” OR “Honduras” OR “Mexico” OR “Nicaragua” OR “Panama” OR “Paraguay” OR “Peru” OR “Dominican Republic” OR “Uruguay” OR “Venezuela”. No date interval filter was applied. Sequences referring to any genetic marker were included, as well as complete genomes.

The sequences were carefully manually reviewed and, once confirmed as being pathogenic leptospires obtained from dogs in Latin America, information on species, serogroup, country of origin, and clinical history was collected. Geographical distribution of the genetic data on Latin America was performed on R-studio (v3.6.1.) using the *rgdal* package.

### 2.2. Phylogenetic Analyses

Sequences from 16S rRNA and *sec*Y genes were selected for phylogenetic analysis due to its more representative number of sequences and a well-defined use for leptospiral genetic characterization [39]. Very short sequences were excluded to avoid bias. After the alignment of sequences on ClustalX v 2.0 software [40], a maximum likelihood (ML) tree was constructed using the Tamura-Nei model (TN92) in MEGA X version 10.0.5 software [41], as it was determined to be the best-fitting model of DNA substitution using the Bayesian information criterion. Genetic distances were calculated using the TN92 model on MEGA X.

For better visualization of haplotype distribution, a haplotype network, a widely used approach for analyzing and visualizing the relationships among DNA sequences within a population or species [42], based on secY sequences was constructed through the population genetics software PopART [43], using the media-joining inference method [44]. The reference sequence *L. interrogans* L1-130 was included in all analyses.

## 3. Results

### 3.1. Description of Genetic Data Obtained

After a careful search in the GenBank platform, a total of 148 sequences were found according to the inclusion criteria adopted (pathogenic *Leptospira* species infecting dogs from Latin America). Summarized information about the sequences included can be found in Table 1 and detailed information is shown in the supplementary material (Table S1).

**Figure 1:**
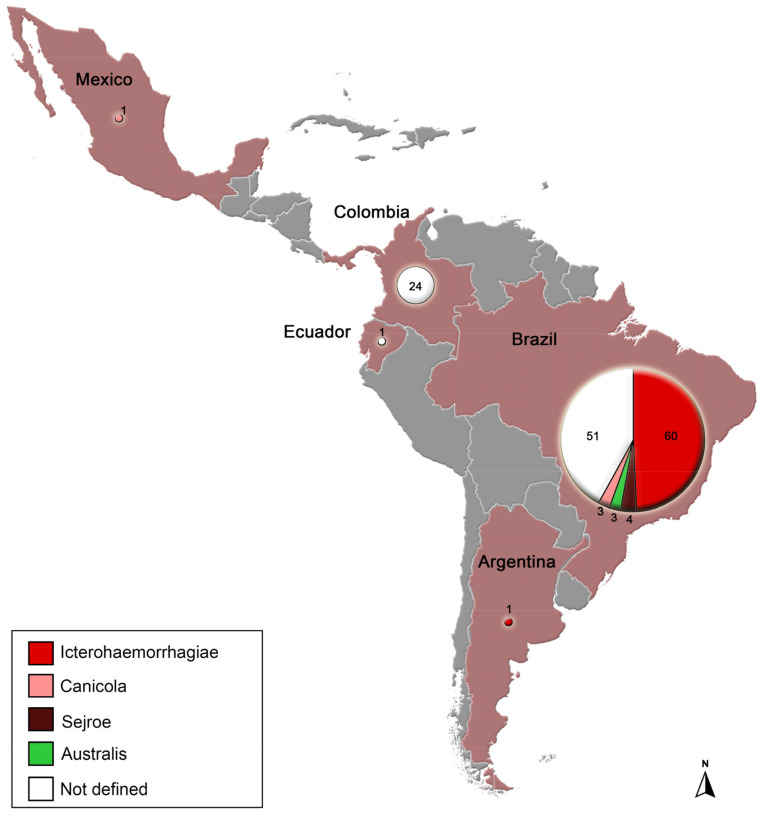
Distribution of pathogenic *Leptospira* spp. From dogs identified by genetic markers according to serogroup in Latin America. Numbers inside circles refer to absolute frequency.

The records included sequences from the genes 16S rRNA (*n* = 80/54%), *sec*Y (*n* = 52/35.1%), *lip*L32 (*n* = 10/6.8%), and *gsp* (*n* = 1/0.6%) and five whole genomes (3.4%). From these, the majority was from Brazil (*n* = 121/81.8%), followed by Colombia (*n* = 24/16.2%), with few reports in Ecuador, Argentina, and Mexico (only one in each, totalizing 3% of the records) (Figure 1).

Three species were identified, *L. interrogans*, the most prevalent (*n* = 138/93.2%), with few reports of *L. santarosai* (*n* = 7/4.8%) and *L. noguchii* (*n* = 3/2%). Serogroups were identified in only 72 strains, with Icterohaemorrhagiae being the most prevalent (*n* = 61/84.7%). Other serogroups identified were Australis (*n* = 3/4.2%), Canicola (*n* = 4/5.5%), and Sejroe (*n* = 4/5.5%). Clinical history of dogs was provided in 83 records, with over half identified as symptomatic (*n* = 53/63.9%) and 30 (36.1%) as asymptomatic.

### 3.2. Molecular Epidemiology Based on Phylogenetic Analyses

Sequences from 16S rRNA and *sec*Y genes were aligned and submitted to genetic analysis, with a cutoff point for the alignments of 258 bp and 393 bp, respectively. A very low genetic distance was observed in both genetic markers (TN92 < 0.01 ± 0.00), reflecting a higher than 99% similarity between sequences.

**Figure 2:**
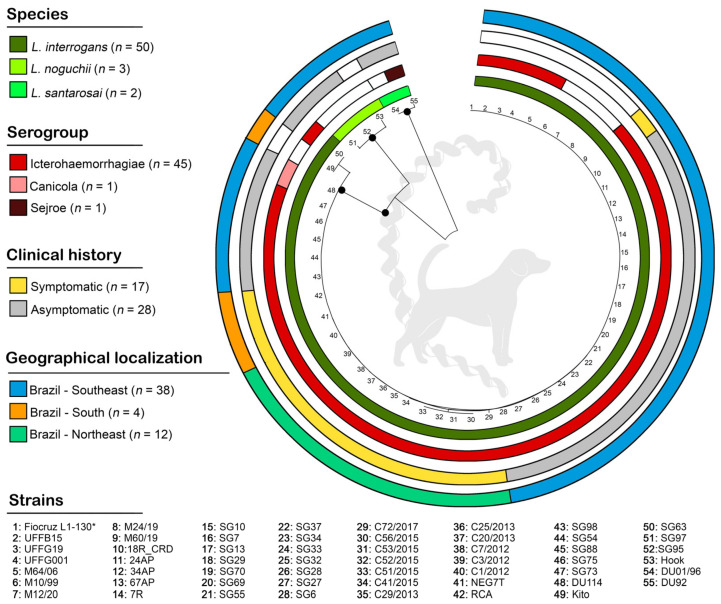
Maximum likelihood phylogenetic tree (1000 bootstrap replicates) based on *sec*Y gene sequences from Latin American dogs. Bootstrap values are shown with the size of circles in the middle of the branches (bootstraps lower than 50 are not shown). From inner to outer circles, the phylogeny is characterized according to species, serogroups, clinical history, and geographical localization, given by colors (white color indicates that the information was not available). * Reference sequence (host = human).

The ML phylogenetic tree based on *sec*Y sequences (*n* = 55) confirmed the three circulating species (*L. interrogans*, *L. noguchii*, and *L. santarosai*), and demonstrated a very homogeneous subcluster in *L. interrogans*, including the reference strain Fiocruz L1-130. No specific clusters were observed according to serogroup, clinical history, or geographical localization, as clearly illustrated in Figure 2.

**Figure 3:**
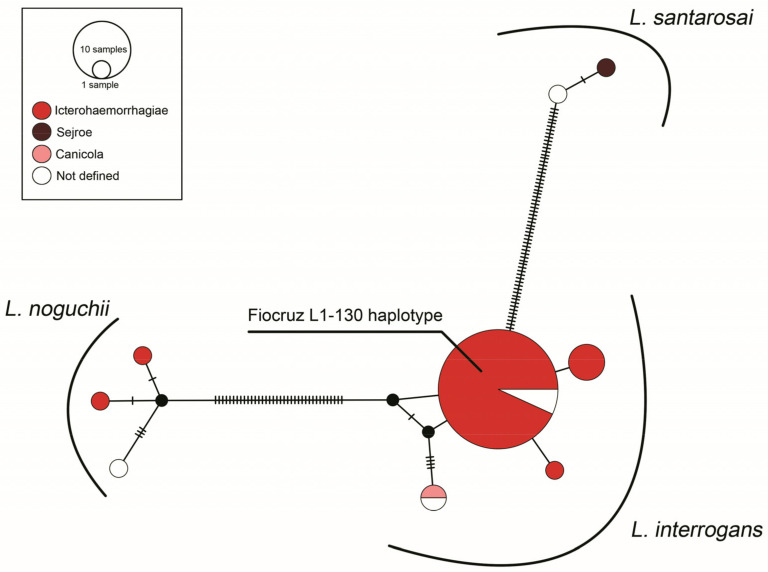
Haplotype network based on *Leptospira* spp. *sec*Y locus (*n* = 56), distributed according serogroup. Area of the circle is proportional to number of sequences. Hatch marks indicate the number of mutations.

The haplotype network demonstrated single haplotypes for the species *L. noguchii* (*n* = 3) and *L. santarosai* (*n* = 2), and four haplotypes in *L. interrogans*, including a major comprising 40 sequences, most were identified as Icterohaemorrhagiae, all identical to the strain Fiocruz L1-130 (Figure 3).

## 4. Discussion

In the present study, we gathered and genetically analyzed the deposited sequences of *Leptospira* spp. identified in dogs from Latin America. We have searched for sequences of all Latin American countries, most of them belonged to Brazilian studies (81.8%). Nevertheless, this outcome does not reflect that Brazil has a higher occurrence of canine leptospirosis than the others. The high quantity of deposited sequences of Brazilian dogs is most probably a mirror of the research effort in this field. Therefore, the study of canine leptospirosis should be extensively performed in other countries.

The *L. interrogans* prevalence in dogs was drastically higher than other leptospiral species, just as observed in humans and rats [45,46]. *L. noguchii* and *L. santarosai*, although less prevalent than *L. interrogans*, were also found herein. In Brazil, a strain of *L. noguchii* serogroup Australis were recovered from a symptomatic male stray dog kidney tissue [47], and two urine samples of asymptomatic dogs were identified as *L. noguchii* by PCR targeting the gene *sec*Y [22]. *L. santarosai* serogroup Sejroe was recovered from the urine of an asymptomatic dog [48]. In Colombia, *L. santarosai* was identified by PCR in one unvaccinated dog’s urine [49]. Thereby, the presence of pathogenic species of *Leptospira* other than *L. interrogans*, although not common*,* cannot be neglected, since they may act as emerging pathogens to dogs. In addition, *L. noguchii* and *L. santarosai* have also been reported in other animal hosts in South America, such as ruminants, opossums, weasels, and armadillos [50,51,52].

In relation to the serogroup distribution, even though only half of the sequences had that information, strains of Icterohaemorrhagiae were by far the major agent of canine infection. In contrast, many serosurveys studies carried out in Latin America frequently reported the presence of strains of Canicola, Autumnalis, and Pomona besides Icterohaemorrhagiae [29,35,36,37,38], and their real role in canine leptospirosis remains to be elucidated. On the other hand, the number of sequences recognized as the Canicola serogroup was surprisingly low since dogs are known to be the maintenance host of this serogroup [10]. Although few sequences of Canicola were deposited, this serogroup plays a major role in serosurveys conducted in South America [49,53,54]. The fact that only 36.1% of the records were identified to be originated from asymptomatic dogs may be responsible for this low rate of Canicola sequences, this number could be higher if more studies were conducted in asymptomatic or chronically infected dogs. Until recently, infections by incidental serogroups, such as Icterohaemorrhagiae, Pomona, and Australis, were linked to severe clinical illness [11,21]. Our outcomes demonstrate that, despite a great number of sequences being recognized as incidental serogroups, almost one-third of the animals were asymptomatic carriers. Those results reinforce previous studies that demonstrated that, besides the classic host-adapted features between dogs and Canicola strains, dogs can also be infected and shed non-adapted strains and remain asymptomatic [22,30,31].

In fact, the high genetic similarity between the sequences of clinically ill and asymptomatic dogs reinforces the hypothesis that the same strain can lead to different clinical outcomes [30]. However, a better understanding of this question with more accurate and definitive answers will only be obtained with advances in genomics, proteomics, and transcriptomics studies, particularly focused on agent–host interactions.

The genetic characterization of the *Leptospira* spp. from dogs is discrete. The absence of a standardized genetic marker for sequencing is an important factor in the gap in the molecular characterization of leptospires. In that context, the use of the *sec*Y gene as a sequencing target has been encouraged, due to its good taxonomic resolution [39]. Other studies have already performed a wide evaluation of deposited leptospiral *sec*Y sequences of other animal species and from different countries [55,56], demonstrating the feasibility of this gene as a target in molecular studies. Although *sec*Y sequencing has been encouraged, in the present study, it represented only 35.1% of the obtained sequences. The target 16S rRNA represented more than half of the sequences; this target was one of the firsts used in *Leptospira* spp. molecular characterization, but its low taxonomic resolution is not useful to differentiate and characterize pathogenic species of the genus [57]. The *lipl*32 gene, representing 6.8% of the sequences, is specific to pathogenic species of *Leptospira* and its sequence is very conserved in *Leptospira* spp., that is why its taxonomic resolution is also low [58]. Nevertheless, even with different gene targets being evaluated, the sequences showed high homology (>99%) between them. It is noteworthy that such similarity suggests the presence of a major well-adapted strain circulating in Latin America.

Regarding the haplotype network, the distribution of the clusters was not influenced by the serogroups, clinical aspects, or the geographic origin of the sequences. The larger haplogroup was composed of more than 40 sequences, all genetically similar to the strain Fiocruz L1-130, (*L. interrogans* serogroup Icterohaemorrhagiae) known as the major agent of human leptospirosis in Brazil [30,59,60,61]. The distribution of those clonal strains of L1-130 by Latin America highlights a ubiquitous circulation pattern and a huge concern to public health.

Concerns about the possibility of infectious disease outbreaks and epidemics have grown during the past few decades [62]. Almost all these illnesses are zoonoses and call for a multidisciplinary approach. These worries led to the development of One Health, a collaborative, multidisciplinary, and cross-sectoral strategy to address the threats from Environment-human relations. WHO defines One Health as “an integrated, unifying approach to balance and optimize the health of people, animals and the environment” [63].

Leptospirosis, as one of the most common zoonotic bacterial infections worldwide, encompasses all aspects of a One Health approach. Its understanding involves in-depth knowledge of the transmission processes, animal reservoir hosts involved, environmental sources of the organism, climate conditions that affect the transmission, and the effects of human occupational activities. Maintaining transmission depends heavily on pathogen characteristics, reservoir host animals, and environmental conditions.

Dogs may act as sentinels of human exposure to leptospirosis [33] and can harbor and shed pathogenic leptospires. Dogs and their owners may share a similar ecological bioaccumulation habitat, and they may serve as sentinels or reservoirs for a variety of zoonotic diseases. Leptospirosis is one of these, and it poses a risk to both humans’ and dogs’ lives because it can seriously affect several organ systems and result in death. Particularly in metropolitan areas, the full impact of dogs on the spread of zoonotic illnesses is unknown. In that context, it is known that asymptomatic dogs can persistently shed leptospires in their urine [22,31]. In Latin America, as most countries in the world, licensed vaccines against canine leptospirosis are bivalent, composed of serovars Icterohaemorrhagiae and Canicola [64]. Although vaccination can reduce urinary shedding, even worldwide recommended protocols of immunization do not prevent kidney colonization and vaccinated dogs can act as carriers of leptospires [65]. Similarly, cats infected with Leptospira spp. remain asymptomatic with very low antibody titres, even after vaccination, reflecting a short-term adaptative immune response that tolerates the infection and contributes with the maintenance of the bacteria in rural and urban areas [66].

Currently, both human and canine species have close proximity and share the same housing space, which qualifies dogs as an important pillar in the epidemiology of zoonotic diseases. Latin American countries have an alarming dense population of stray or free-roaming dogs [67] that allied to the tropical environment and high endemicity, can play a crucial role in leptospirosis transmission to humans. Still, little is known about the real impact of the closeness of the canines with humans or the high presence of stray dogs with human leptospirosis features. However, the association of asymptomatic shedding and the infection by strains with high zoonotic potential puts canine leptospirosis as a hazard to One Health.

In this context, the findings of the present study, that strains obtained from dogs in Latin America are essentially the same that determine severe disease in humans and circulates in other domestic and wild species deserves attention. Specifically in the case of dogs and humans, both species share the same environment and epidemiological aspects, reinforce the role of the need for a One Health approach in order to reduce both animal and human cases simultaneously. They are interdependent and, particularly in urban settings where they share the same sanitary conditions and are exposed to the same rodent population, it is not possible to consider the study or the understanding and control of leptospirosis without considering a broad approach to it.

## 5. Conclusions

We described the molecular epidemiology of leptospires circulating among dogs in Latin America and demonstrated a very genetically homogeneous group, with a predominance of a larger haplogroup that includes identical sequences to *L. interrogans* Fiocruz L1-130, elucidating its ubiquitous circulation pattern and alerting to the important role of dogs in the One Health transmission dynamics of leptospirosis.

## Figures and Tables

**Table 1:** Summarized information about pathogenic *Leptospira* spp. identified through genetic sequencing in dogs from Latin America.

Country	Species	Strain/Sample ID	Clinical History	Serogroup
Argentina (*n* = 1)	*L. interrogans* (*n* = 1)	28	symptomatic	Icterohaemorrhagiae
Brazil (*n* = 105)	*L. interrogans* (*n* = 99)	18R_CRD, 7R, C1/2012, C20/2013, C25/2013, C29/2013, C3/2012, C41/2015, C51/2015, C52/2015, C53/2015, C56/2015, C7/2012, C72/2017, Dog 10, Dog 16, Dog 2, Dog 9, NEG7, RCA	symptomatic	Icterohaemorrhagiae
		24AP, 34AP, 67AP, SG10, SG13, SG27, SG28, SG29, SG3, SG32, SG33, SG34 SG37, SG54, SG55, SG6, SG69, SG7, SG70, SG73, SG75, SG88, SG98	asymptomatic	
		M10/99, M64/06, UFFB15, UFFG001, UFFG19	unknown	
		C80/2017, Dog 6	symptomatic	Canicola
		DU114	asymptomatic	Canicola
		Dog 25, Dog 26, Dog 8	symptomatic	Australis
		Dog 13, Dog 30, Dog 4, Dog 5	symptomatic	N/D
		01/014, 01/017, 01/037, 01/070, 01/072, 01/077, 01/078, 01/089, 01/095, 07/070, 13R, 14F, 15F, 16F, 17F, 18F, 21F, 22F, DUZO, H05, H07, H08, H09, H10, H18, H20, H24, H30, H33, H41, H43, H44, Kito, M12/20, M24/19, M60/19, USPIA USPIB, USPIC, USPID, USPIIA	unknown	N/D
	*L. noguchii* (*n* = 3)	SG95, SG97	asymptomatic	N/D
		Hook	unknown	N/D
	*L. santarosai* (*n* = 3)	DU92, DUPA	asymptomatic	Sejroe
		DU01/96	asymptomatic	N/D
Colombia (*n* = 17)	*L. interrogans* (*n* = 17)	CfCASol-110-Or, CfCASol-112-Or, CfCASol-135-Or, CfCASol-37-Or, CfCASol-40-As, CfCASol-53-As, CfCASol-59-Or, CfCASol-61-A, CfCASol-72-As, RCA	symptomatic	N/D
		CfCASol-23-Or, CfCASol-34-O, CfCASol-36-Or, Dog 133, Dog 146, Dog 167, Dog 196	unknown	N/D
Ecuador (*n* = 1)	*L.* *santarosai*	Calderón-1	unknown	N/D
Mexico (*n* = 1)	*L. interrogans*	LOCaS46	asymptomatic	Canicola

N/D: Not defined.

## Data Availability

Not applicable.

## Supplementary Materials

The following supporting information can be downloaded at: https://www.mdpi.com/article/10.3390/ani13152422/s1, Table S1: Detailed information about pathogenic *Leptospira* spp. identified through genetic sequencing in dogs from Latin America. Strains are presented in alphabetical order.

## References

[1] Costa F., Hagan J.E., Calcagno J., Kane M., Torgerson P., Martinez-Silveira M.S., Stein C., Abela-Ridder B., Ko A.I. (2015). Global Morbidity and Mortality of Leptospirosis: A Systematic Review. PLoS Negl. Trop. Dis..

[2] Cameron C.E. (2015). Leptospiral Structure, Physiology, and Metabolism. Curr. Top. Microbiol. Immunol..

[3] Nakamura S. (2022). Motility of the Zoonotic Spirochete *Leptospira*: Insight into Association with Pathogenicity. Int. J. Mol. Sci..

[4] Samrot A.V., Sean T.C., Bhavya K.S., Sahithya C.S., Chan-Drasekaran S., Palanisamy R., Robinson E.R., Subbiah S.K., Mok P.L. (2021). Leptospiral Infection, Pathogenesis and Its Diagnosis—A Review. Pathogens.

[5] Levett P.N. (2015). Systematics of *Leptospiraceae*. Curr. Top. Microbiol. Immunol..

[6] Vincent A.T., Schiettekatte O., Goarant C., Neela V.K., Bernet E., Thibeaux R., Ismail N., Mohd Khalid M.K.N., Amran F., Masuzawa T. (2019). Revisiting the Taxonomy and Evolution of Pathogenicity of the Genus Leptospira through the Prism of Genomics. PLoS Negl. Trop. Dis..

[7] Korba A.A., Lounici H., Kainiu M., Vincent A.T., Mariet J.-F., Veyrier F.J., Goarant C., Picardeau M. (2021). *Leptospira ainlahdjerensis* Sp. Nov., *Leptospira ainazelensis* Sp. Nov., *Leptospira abararensis* Sp. Nov. and *Leptospira chreensis* Sp. Nov., Four New Species Isolated from Water Sources in Algeria. Int. J. Syst. Evol. Microbiol..

[8] Zhang C., Yang H., Li X., Cao Z., Zhou H., Zeng L., Xu J., Xu Y., Chang Y.-F., Guo X. (2015). Molecular Typing of Pathogenic *Leptospira* Serogroup Icterohaemorrhagiae Strains Circulating in China during the Past 50 Years. PLoS Negl. Trop. Dis..

[9] Ko A.I., Goarant C., Picardeau M. (2009). *Leptospira*: The Dawn of the Molecular Genetics Era for an Emerging Zoonotic Pathogen. Nat. Rev. Microbiol..

[10] Ellis W.A. (2015). Animal Leptospirosis. Curr. Top. Microbiol. Immunol..

[11] Santos C.M., Dias G.C.D.R.S., Saldanha A.V.P., Esteves S.B., Cortez A., Guedes I.B., Heinemann M.B., Gonçales A.P., Miotto B.A. (2021). Molecular and Serological Characterization of Pathogenic *Leptospira* Spp. Isolated from Symptomatic Dogs in a Highly Endemic Area, Brazil. BMC Vet. Res..

[12] Haake D.A., Levett P.N. (2015). Leptospirosis in Humans. Curr. Top. Microbiol. Immunol..

[13] Santos N.J., Sousa E., Reis M.G., Ko A.I., Costa F. (2017). Rat Infestation Associated with Environmental Deficiencies in an Urban Slum Community with High Risk of Leptospirosis Transmission. Cad. Saúde Pública.

[14] Adler B., de la Peña Moctezuma A. (2010). *Leptospira* and Leptospirosis. Vet. Microbiol..

[15] Reagan K.L., Sykes J.E. (2019). Diagnosis of Canine Leptospirosis. Vet. Clin. N. Am. Small Anim. Pract..

[16] Putz E.J., Nally J.E. (2020). Investigating the Immunological and Biological Equilibrium of Reservoir Hosts and Pathogenic *Leptospira*: Balancing the Solution to an Acute Problem?. Front. Microbiol..

[17] Adler B., Lo M., Seemann T., Murray G.L. (2011). Pathogenesis of Leptospirosis: The Influence of Genomics. Vet. Microbiol..

[18] Schuller S., Francey T., Hartmann K., Hugonnard M., Kohn B., Nally J.E., Sykes J. (2015). European Consensus Statement on Leptospirosis in Dogs and Cats. J. Small Anim. Pract..

[19] Klarenbeek A., Schuffner W.A.P. (1933). Appearance in Holland of Leptospira Differing from Weil Strain. Ned. Tijdschr. Geneeskd..

[20] Azócar-Aedo L., Monti G. (2016). Meta-Analyses of Factors Associated with Leptospirosis in Domestic Dogs. Zoonoses Public Health.

[21] Pinto P.S., Libonati H., Lilenbaum W. (2017). A Systematic Review of Leptospirosis on Dogs, Pigs, and Horses in Latin America. Trop. Anim. Health Prod..

[22] Sant’Anna da Costa R., Di Azevedo M.I.N., Dos Santos Baptista Borges A.L., Carvalho-Costa F.A., Martins G., Lilenbaum W. (2021). Persistent High Leptospiral Shedding by Asymptomatic Dogs in Endemic Areas Triggers a Serious Public Health Concern. Animals.

[23] Major A., Schweighauser A., Francey T. (2014). Increasing Incidence of Canine Leptospirosis in Switzerland. Int. J. Environ. Res. Public Health.

[24] Sykes J.E. (2012). Chapter 42: Leptospirosis. Infectious Diseases of the Dog and Cat.

[25] Raj J., Campbell R., Tappin S. (2021). Clinical Findings in Dogs Diagnosed with Leptospirosis in England. Vet. Rec..

[26] Lippi I., Puccinelli C., Perondi F., Ceccherini G., Pierini A., Marchetti V., Citi S. (2021). Predictors of Fatal Pulmonary Haemorrhage in Dogs Affected by Leptospirosis Approaching Haemodialysis. Vet. Sci..

[27] Greenlee J.J., Bolin C.A., Alt D.P., Cheville N.F., Andreasen C.B. (2004). Clinical and Pathologic Comparison of Acute Leptospirosis in Dogs Caused by Two Strains of *Leptospira kirschneri* Serovar Grippotyphosa. Am. J. Vet. Res..

[28] Greenlee J.J., Alt D.P., Bolin C.A., Zuerner R.L., Andreasen C.B. (2005). Experimental Canine Leptospirosis Caused by *Leptospira interrogans* Serovars Pomona and Bratislava. Am. J. Vet. Res..

[29] Esteves S.B., Santos C.M., Silva B.C.S., Salgado F.F., Guilloux A.G.A., Cortez A., Lucco R.C., Miotto B.A. (2023). Time for Change? A Systematic Review with Meta-Analysis of Leptospires Infecting Dogs to Assess Vaccine Compatibility in Brazil. Prev. Vet. Med..

[30] Di Azevedo M.I.N., Santanna R., Carvalho-Costa F.A., Lilenbaum W. (2022). The Same Strain Leading to Different Clinical Outcomes: The Enigma behind the Canine Leptospirosis. Microb. Pathog..

[31] Miotto B.A., Guilloux A.G.A., Tozzi B.F., Moreno L.Z., da Hora A.S., Dias R.A., Heinemann M.B., Moreno A.M., de Souza Filho A.F., Lilenbaum W. (2018). Prospective Study of Canine Leptospirosis in Shelter and Stray Dog Populations: Identification of Chronic Carriers and Different *Leptospira* Species Infecting Dogs. PloS ONE.

[32] do Couto A.C., Gravinatti M.L., Pellizzaro M., Kmetiuk L.B., Yamakawa A.C., da Silva E.C., Felipetto L.G., Langoni H., de Souza Leandro A., de Santi C.E. (2022). One Health Approach on Serosurvey of Anti-*Leptospira* Spp. in Homeless Persons and Their Dogs in South Brazil. One Health.

[33] Pilau N.N., Lubar A.A., Daneji A.I., Mera U.M., Magaji A.A., Abiayi E.A., Chaiboonma K.L., Busayo E.I., Vinetz J.M., Matthias M.A. (2022). Serological and Molecular Epidemiology of Leptospirosis and the Role of Dogs as Sentinel for Human Infection in Nigeria. Heliyon.

[34] Flores B.J., Pérez-Sánchez T., Fuertes H., Sheleby-Elías J., Múzquiz J.L., Jirón W., Duttmann C., Halaihel N. (2017). A Cross-Sectional Epidemiological Study of Domestic Animals Related to Human Leptospirosis Cases in Nicaragua. Acta Trop..

[35] Cárdenas N.C., Infante G.P., Pacheco D.A.R., Diaz J.P.D., Wagner D.C.M., Dias R.A., Neto J.S.F., Amaku M., Vargas-Pinto P., Polo L. (2018). Seroprevalence of *Leptospira* Spp. Infection and Its Risk Factors among Domestic Dogs in Bogotá, Colombia. Vet. Anim. Sci..

[36] Azócar-Aedo L., Monti G. (2022). Seroprevalence of Pathogenic *Leptospira* Spp. in Domestic Dogs from Southern Chile and Risk Factors Associated with Different Environments. Prev. Vet. Med..

[37] Azócar-Aedo L. (2023). Basic Aspects and Epidemiological Studies on Leptospirosis Carried Out in Animals in Chile: A Bibliographic Review. Trop. Med. Infect. Dis..

[38] Grune Loffler S., Passaro D., Samartino L., Soncini A., Romero G., Brihuega B. (2014). Genotypes of *Leptospira* Spp. Strains Isolated from Dogs in Buenos Aires, Argentina. Rev. Argent. Microbiol..

[39] Di Azevedo M.I.N., Lilenbaum W. (2021). An Overview on the Molecular Diagnosis of Animal Leptospirosis. Lett. Appl. Microbiol..

[40] Larkin M.A., Blackshields G., Brown N.P., Chenna R., McGettigan P.A., McWilliam H., Valentin F., Wallace I.M., Wilm A., Lopez R. (2007). Clustal W and Clustal X Version 2.0. Bioinformatics.

[41] Kumar S., Stecher G., Li M., Knyaz C., Tamura K. (2018). MEGA X: Molecular Evolutionary Genetics Analysis across Computing Platforms. Mol. Biol. Evol..

[42] Paradis E. (2018). Analysis of Haplotype Networks: The Randomized Minimum Spanning Tree Method. Methods Ecol. Evol..

[43] Leigh J.W., Bryant D. (2015). Monte Carlo Strategies for Selecting Parameter Values in Simulation Experiments. Syst. Biol..

[44] Bandelt H.J., Forster P., Röhl A. (1999). Median-Joining Networks for Inferring Intraspecific Phylogenies. Mol. Biol. Evol..

[45] Guernier V., Richard V., Nhan T., Rouault E., Tessier A., Musso D. (2017). Leptospira Diversity in Animals and Humans in Tahiti, French Polynesia. PLoS Negl. Trop. Dis..

[46] López-Osorio S., Molano D.A., López-Arias A., Rodríguez-Osorio N., Zambrano C., Chaparro-Gutiérrez J.J. (2022). Seroprevalence and Molecular Characterization of *Leptospira* Spp. in Rats Captured near Pig Farms in Colombia. Int. J. Environ. Res. Public Health.

[47] Silva E.F., Cerqueira G.M., Seyffert N., Seixas F.K., Hartwig D.D., Athanazio D.A., Pinto L.S., Queiroz A., Ko A.I., Brod C.S. (2009). *Leptospira noguchii* and Human and Animal Leptospirosis, Southern Brazil. Emerg. Infect. Dis..

[48] Miotto B.A., Moreno L.Z., Guilloux A.G.A., de Sousa G.O., Loureiro A.P., Moreno A.M., Lilenbaum W., Vasconcellos S.A., Heinemann M.B., Hagiwara M.K. (2016). Molecular and Serological Characterization of the First *Leptospira santarosai* Strain Isolated from a Dog. Acta Trop..

[49] Perez-Garcia J., Monroy F.P., Agudelo-Florez P. (2022). Canine Leptospirosis in a Northwestern Region of Colombia: Serological, Molecular and Epidemiological Factors. Pathogens.

[50] Zarantonelli L., Suanes A., Meny P., Buroni F., Nieves C., Salaberry X., Briano C., Ashfield N., Da Silva Silveira C., Dutra F. (2018). Isolation of Pathogenic *Leptospira* Strains from Naturally Infected Cattle in Uruguay Reveals High Serovar Diversity, and Uncovers a Relevant Risk for Human Leptospirosis. PLoS Negl. Trop. Dis..

[51] Loureiro A.P., Jaeger L.H., Di Azevedo M.I.N., Miraglia F., Moreno L.Z., Moreno A.M., Pestana C.P., Carvalho-Costa F.A., Medeiros M.A., Lilenbaum W. (2020). Molecular Epidemiology of *Leptospira noguchii* Reveals Important Insights into a One Health Context. Transbound. Emerg. Dis..

[52] Aymée L., Di Azevedo M.I.N., Borges A.L.D.S.B., Carvalho-Costa F.A., Lilenbaum W. (2022). *Leptospira* Spp. Strains Associated with Bovine Genital Leptospirosis (BGL). Microb. Pathog..

[53] Lelu M., Muñoz-Zanzi C., Higgins B., Galloway R. (2015). Seroepidemiology of Leptospirosis in Dogs from Rural and Slum Communities of Los Rios Region, Chile. BMC Vet. Res..

[54] Benitez A.D.N., Monica T.C., Miura A.C., Romanelli M.S., Giordano L.G.P., Freire R.L., Mitsuka-Breganó R., Martins C.M., Biondo A.W., Serrano I.M. (2020). Spatial and Simultaneous Seroprevalence of Anti-*Leptospira* Antibodies in Owners and Their Domiciled Dogs in a Major City of Southern Brazil. Front. Vet. Sci..

[55] Barragan V.A., Mejia M.E., Trávez A., Zapata S., Hartskeerl R.A., Haake D.A., Trueba G.A. (2011). Interactions of *Leptospira* with Environmental Bacteria from Surface Water. Curr. Microbiol..

[56] Torres F.D., Borges A.L.D.S.B., Kolesnikovas C., Domit C., Barbosa C.B., Carvalho-Costa F.A., Di Azevedo M.I.N., Lilenbaum W. (2023). Pinnipeds Carriers of Pathogenic *Leptospira*: New Data Based on Molecular Characterization. Res. Vet. Sci..

[57] Caimi K., Ruybal P. (2020). *Leptospira* Spp., a Genus in the Stage of Diversity and Genomic Data Expansion. Infect. Genet. Evol. J. Mol. Epidemiol. Evol. Genet. Infect. Dis..

[58] Kumaran S.K., Bakar M.F.A., Mohd-Padil H., Mat-Sharani S., Sakinah S., Poorani K., Alsaeedy H., Peli A., Wei T.S., Ling M.P. (2017). 3D Modelling of the Pathogenic *Leptospira* Protein LipL32: A Bioinformatics Approach. Acta Trop..

[59] Jaeger L.H., Pestana C.P., Carvalho-Costa F.A., Medeiros M.A., Lilenbaum W. (2018). Characterization of the Clonal Subpopulation Fiocruz L1-130 of *Leptospira interrogans* in Rats and Dogs from Brazil. J. Med. Microbiol..

[60] Miraglia F., Matsuo M., Morais Z.M., Dellagostin O.A., Seixas F.K., Freitas J.C., Hartskeerl R., Moreno L.Z., Costa B.L., Souza G.O. (2013). Molecular Characterization, Serotyping, and Antibiotic Susceptibility Profile of *Leptospira interrogans* Serovar Copenhageni Isolates from Brazil. Diagn. Microbiol. Infect. Dis..

[61] Pereira M.M., Matsuo M.G., Bauab A.R., Vasconcelos S.A., Moraes Z.M., Baranton G., Saint Girons I. (2000). A Clonal Subpopulation of *Leptospira interrogans* Sensu Stricto Is the Major Cause of Leptospirosis Outbreaks in Brazil. J. Clin. Microbiol..

[62] Pham H.T., Tran M.-H. (2022). One Health: An Effective and Ethical Approach to Leptospirosis Control in Australia. Trop. Med. Infect. Dis..

[63] One Health. https://www.who.int/news-room/questions-and-answers/item/one-health.

[64] Klaasen H.L.B.M., Molkenboer M.J.C.H., Vrijenhoek M.P., Kaashoek M.J. (2003). Duration of Immunity in Dogs Vaccinated against Leptospirosis with a Bivalent Inactivated Vaccine. Vet. Microbiol..

[65] Sant’Anna da Costa R., Di Azevedo M.I.N., Dos Santos Baptista Borges A.L., Aymée L., Martins G., Lilenbaum W. (2022). Effect of Vaccination against *Leptospira* on Shelter Asymptomatic Dogs Following a Long-Term Study. Animals.

[66] Murillo A., Cuenca R., Serrano E., Marga G., Ahmed A., Cervantes S., Caparrós C., Vieitez V., Ladina A., Pastor J. (2020). *Leptospira* Detection in Cats in Spain by Serology and Molecular Techniques. Int. J. Environ. Res. Public Health.

[67] Mota-Rojas D., Calderón-Maldonado N., Lezama-García K., Sepiurka L., Maria Garcia R.C. (2021). Abandonment of Dogs in Latin America: Strategies and Ideas. Vet. World.

