# Supplementary material for: Molecular Epidemiology of Pathogenic Leptospira spp. Infecting Dogs in Latin America
Source: Animals (Basel). 2023 Jul 27;13(15):2422. doi: 10.3390/ani13152422 (PMC10417440; doi:10.3390/ani13152422)
Supplement: Supplementary file 1 [file animals-13-02422-s001.zip › animals-2476982-supplementary.pdf]

Supplementary Table S1: Detailed information about pathogenic *Leptospira* spp. identified through genetic sequencing in dogs from Latin America. Strains are presented in alphabetical order.

Strain (Accession number)	Genetic marker	Country	Species	Serogroup	Clinical history	Year of identification	Reference
01/014 (KX891325)	16S rRNA	Brazil	*L. interrogans*	N/D	Unknown	2016	[41]
01/017 (KX891326)	16S rRNA	Brazil	*L. interrogans*	N/D	Unknown	2016	[41]
01/037 (KX891327)	16S rRNA	Brazil	*L. interrogans*	N/D	Unknown	2016	[41]
01/070 (KX891328)	16S rRNA	Brazil	*L. interrogans*	N/D	Unknown	2016	[41]
01/072 (KX891330)	16S rRNA	Brazil	*L. interrogans*	N/D	Unknown	2016	[41]
01/077 (KX891331)	16S rRNA	Brazil	*L. interrogans*	N/D	Unknown	2016	[41]
01/078 (KX891332)	16S rRNA	Brazil	*L. interrogans*	N/D	Unknown	2016	[41]
01/089 (KX891333)	16S rRNA	Brazil	*L. interrogans*	N/D	Unknown	2016	[41]
01/095 (KX891334)	16S rRNA	Brazil	*L. interrogans*	N/D	Unknown	2016	[41]
07/070 (KX891329)	16S rRNA	Brazil	*L. interrogans*	N/D	Unknown	2016	[41]
13R (MN114056)	16S rRNA	Brazil	*L. interrogans*	N/D	Unknown	2019	NP
14F (MN114057)	16S rRNA	Brazil	*L. interrogans*	N/D	Unknown	2019	NP
15F (MN114058)	16S rRNA	Brazil	*L. interrogans*	N/D	Unknown	2019	NP
16F (MN114059)	16S rRNA	Brazil	*L. interrogans*	N/D	Unknown	2019	NP
17F (MN11406)	16S rRNA	Brazil	*L. interrogans*	N/D	Unknown	2019	NP
18F (MN114061)	16S rRNA	Brazil	*L. interrogans*	N/D	Unknown	2019	NP
18R_CRD (MZ773515)	secY	Brazil	*L. interrogans*	Ictero*	symptomatic	2018	[25]
21F (MN114062)	16S rRNA	Brazil	*L. interrogans*	N/D	Unknown	2019	NP
22F (MN114063)	16S rRNA	Brazil	*L. interrogans*	N/D	Unknown	2019	NP
24AP (MZ773516)	*sec*Y	Brazil	*L. interrogans*	Ictero*	asymptomatic	2018	[25]
28 (KF184514)	*Lip*L32	Argentina	*L. interrogans*	Ictero*	symptomatic	2013	[42]
34AP (MZ773517)	*sec*Y	Brazil	*L. interrogans*	Ictero*	asymptomatic	2018	[25]
67AP (MZ773518)	*sec*Y	Brazil	*L. interrogans*	Ictero*	asymptomatic	2018	[25]
7R (MZ773514)	*sec*Y	Brazil	*L. interrogans*	Ictero*	symptomatic	2018	[25]
C1/2012 (KX058885)	16S rRNA	Brazil	*L. interrogans*	Ictero*	symptomatic	2016	[43]
C1/2012 (MN394902)	*sec*Y	Brazil	*L. interrogans*	Ictero*	symptomatic	2016	[43]
C20/2013 (KX058888)	16S rRNA	Brazil	*L. interrogans*	Ictero*	symptomatic	2016	[43]
C20/2013 (MN394905	*sec*Y	Brazil	*L. interrogans*	Ictero*	symptomatic	2016	[43]
C25/2013 (KX058889)	16S rRNA	Brazil	*L. interrogans*	Ictero*	symptomatic	2016	[43]
C25/2013 (MN394906)	*sec*Y	Brazil	*L. interrogans*	Ictero*	symptomatic	2016	[43]
C29/2013 (KX058890)	16S rRNA	Brazil	*L. interrogans*	Ictero*	symptomatic	2016	[43]
C29/2013 (MN394907)	*sec*Y	Brazil	*L. interrogans*	Ictero*	symptomatic	2016	[43]
C3/2012 (KX058886)	16S rRNA	Brazil	*L. interrogans*	Ictero*	symptomatic	2016	[43]
C3/2012 (MN394903)	*sec*Y	Brazil	*L. interrogans*	Ictero*	symptomatic	2016	[43]
C41/2015 (MK330631)	16S rRNA	Brazil	*L. interrogans*	Ictero*	symptomatic	2016	[43]
C41/2015 (MN394908)	*sec*Y	Brazil	*L. interrogans*	Ictero*	symptomatic	2016	[43]
C51/2015 (MK330632)	16S rRNA	Brazil	*L. interrogans*	Ictero*	symptomatic	2016	[43]
C51/2015 (MN394909)	*sec*Y	Brazil	*L. interrogans*	Ictero*	symptomatic	2016	[43]
C52/2015 (MK330633)	16S rRNA	Brazil	*L. interrogans*	Ictero*	symptomatic	2016	[43]
C52/2015 (MN394910)	*sec*Y	Brazil	*L. interrogans*	Ictero*	symptomatic	2016	[43]
C53/2015 (MK330634)	16S rRNA	Brazil	*L. interrogans*	Ictero*	symptomatic	2016	[43]
C53/2015 (MN394911)	*sec*Y	Brazil	*L. interrogans*	Ictero*	symptomatic	2016	[43]
C56/2015 (MK330635)	16S rRNA	Brazil	*L. interrogans*	Ictero*	symptomatic	2016	[43]
C56/2015 (MN394912)	*sec*Y	Brazil	*L. interrogans*	Ictero*	symptomatic	2016	[43]
C7/2012 (KX058887)	16S rRNA	Brazil	*L. interrogans*	Ictero*	symptomatic	2016	[43]
C7/2012 (MN394904)	*sec*Y	Brazil	*L. interrogans*	Ictero*	symptomatic	2016	[43]
C72/2017 (MK330636)	16S rRNA	Brazil	*L. interrogans*	Ictero*	symptomatic	2016	[43]
C72/2017 (MN394913)	*sec*Y	Brazil	*L. interrogans*	Ictero*	symptomatic	2016	[43]
C80/2017 (MT614189)	*sec*Y	Brazil	*L. interrogans*	Canicola	symptomatic	2016	[43]
Calderón-1 (HQ709387)	16S rRNA	Ecuador	*L. santarosai*	N/D	Unknown	2010	[44]
CfCASol-110-Or (MN482700)	16S rRNA	Colombia	*L. interrogans*	N/D	symptomatic	2019	NP
CfCASol-112-Or (MN482699)	16S rRNA	Colombia	*L. interrogans*	N/D	symptomatic	2019	NP

Strain (Accession number)	Genetic marker	Country	Species	Serogroup	Clinical history	Year of identification	Reference
CfCASol-135-Or (MN482698)	16S rRNA	Colombia	*L. interrogans*	N/D	symptomatic	2019	NP
CfCASol-23-Or (MN486060)	*Lip*L32	Colombia	*L. interrogans*	N/D	Unknown	2019	NP
CfCASol-34-As (MN486062)	*Lip*L32	Colombia	*L. interrogans*	N/D	Unknown	2019	NP
CfCASol-34-Or (MN482697)	16S rRNA	Colombia	*L. interrogans*	N/D	symptomatic	2019	NP
CfCASol-34-Or (MN486061)	*Lip*L32	Colombia	*L. interrogans*	N/D	Unknown	2019	NP
CfCASol-36-Or (MN486063)	*Lip*L32	Colombia	*L. interrogans*	N/D	Unknown	2019	NP
CfCASol-37-As (MN486065)	*Lip*L32	Colombia	*L. interrogans*	N/D	Unknown	2019	NP
CfCASol-37-Or (MN482696)	16S rRNA	Colombia	*L. interrogans*	N/D	symptomatic	2019	NP
CfCASol-37-Or (MN486064)	*Lip*L32	Colombia	*L. interrogans*	N/D	Unknown	2019	NP
CfCASol-40-As (MN482695)	16S rRNA	Colombia	*L. interrogans*	N/D	symptomatic	2019	NP
CfCASol-40-As (MN486067)	*Lip*L32	Colombia	*L. interrogans*	N/D	Unknown	2019	NP
CfCASol-40-OR (MN48606)	*Lip*L32	Colombia	*L. interrogans*	N/D	Unknown	2019	NP
CfCASol-53-As (MN482694)	16S rRNA	Colombia	*L. interrogans*	N/D	symptomatic	2019	NP
CfCASol-59-Or (MN482692)	16S rRNA	Colombia	*L. interrogans*	N/D	symptomatic	2019	NP
CfCASol-61-Or (MN486068)	*Lip*L32	Colombia	*L. interrogans*	N/D	Unknown	2019	NP
CfCASol-61-Sa (MN482691)	16S rRNA	Colombia	*L. interrogans*	N/D	symptomatic	2019	NP
CfCASol-72-As (MN482690)	16S rRNA	Colombia	*L. interrogans*	N/D	symptomatic	2019	NP
Dog 10 (MW263936)	16S rRNA	Brazil	*L. interrogans*	Ictero*	symptomatic	2020	[12]
Dog 13 (MW263937)	16S rRNA	Brazil	*L. interrogans*	N/D	symptomatic	2020	[12]
Dog 133 (OP037101)	16S rRNA	Colombia	*L. interrogans*	N/D	Unknown	2022	NP
Dog 146 (OP037102)	16S rRNA	Colombia	*L. interrogans*	N/D	Unknown	2022	NP
Dog 16 (MW263938)	16S rRNA	Brazil	*L. interrogans*	Ictero*	symptomatic	2020	[12]
Dog 167 (OP037103)	16S rRNA	Colombia	*L. interrogans*	N/D	Unknown	2022	NP
Dog 196 (OP037104)	16S rRNA	Colombia	*L. interrogans*	N/D	Unknown	2022	NP
Dog 2 (MW263930)	16S rRNA	Brazil	*L. interrogans*	Ictero*	symptomatic	2020	[12]
Dog 25 (MW263939)	16S rRNA	Brazil	*L. interrogans*	Australis	symptomatic	2020	[12]
Dog 26 (MW263940)	16S rRNA	Brazil	*L. interrogans*	Australis	symptomatic	2020	[12]
Dog 30 (MW263941)	16S rRNA	Brazil	*L. interrogans*	N/D	symptomatic	2020	[12]
Dog 4 (MW263931)	16S rRNA	Brazil	*L. interrogans*	N/D	symptomatic	2020	[12]
Dog 5 (MW263932)	16S rRNA	Brazil	*L. interrogans*	N/D	symptomatic	2020	[12]
Dog 6 (MW263933)	16S rRNA	Brazil	*L. interrogans*	Canicola	symptomatic	2020	[12]
Dog 8 (MW263934)	16S rRNA	Brazil	*L. interrogans*	Australis	symptomatic	2020	[12]
Dog 9 (MW263935)	16S rRNA	Brazil	*L. interrogans*	Ictero*	symptomatic	2020	[12]
DU01/96 (KX008568)	16S rRNA	Brazil	*L. santarosai*	N/D	Unknown	2016	NP
DU01/96 (KX026948)	*secY*	Brazil	*L. santarosai*	N/D	asymptomatic	2016	[26]
DU114 (CP022883)	WGS	Brazil	*L. interrogans*	Canicola	asymptomatic	2017	[47]
DU92 (KU682051)	16S rRNA	Brazil	*L. santarosai*	Sejroe	Unknown	2016	[40]
DU92 (KU682052)	*secY*	Brazil	*L. santarosai*	Sejroe	asymptomatic	2016	[40]
DUPA (KX008567)	16S rRNA	Brazil	*L. santarosai*	Sejroe	Unknown	2016	[26]
DUPA (KX026949)	*secY*	Brazil	*L. santarosai*	Sejroe	asymptomatic	2016	[26]
DUZO (KX008569)	16S rRNA	Brazil	*L. interrogans*	N/D	Unknown	2016	[40]
DUZO (KX026950)	*secY*	Brazil	*L. interrogans*	N/D	Unknown	2016	[26]
H05 (MF599537)	16S rRNA	Brazil	*L. interrogans*	N/D	Unknown	2017	[41]
H07 (MF599538)	16S rRNA	Brazil	*L. interrogans*	N/D	Unknown	2017	[41]
H08 (MF599539)	16S rRNA	Brazil	*L. interrogans*	N/D	Unknown	2017	[41]
H09 (MF599540)	16S rRNA	Brazil	*L. interrogans*	N/D	Unknown	2017	[41]
H10 (MF599541)	16S rRNA	Brazil	*L. interrogans*	N/D	Unknown	2017	[41]
H18 (MF599542)	16S rRNA	Brazil	*L. interrogans*	N/D	Unknown	2017	[41]
H20 (MF599543)	16S rRNA	Brazil	*L. interrogans*	N/D	Unknown	2017	[41]
H24 (MF599544)	16S rRNA	Brazil	*L. interrogans*	N/D	Unknown	2017	[41]
H30 (MF599545)	16S rRNA	Brazil	*L. interrogans*	N/D	Unknown	2017	[41]
H33 (MF599546)	16S rRNA	Brazil	*L. interrogans*	N/D	Unknown	2017	[41]
H41 (MF599547)	16S rRNA	Brazil	*L. interrogans*	N/D	Unknown	2017	[41]
H43 (MG640117)	16S rRNA	Brazil	*L. interrogans*	N/D	Unknown	2017	[41]
H44 (MG640121)	16S rRNA	Brazil	*L. interrogans*	N/D	Unknown	2017	[41]
Hook (AOUC01000111)	WGS	Brazil	*L. noguchii*	N/D	Unknown	2013	NP

Strain (Accession number)	Genetic marker	Country	Species	Serogroup	Clinical history	Year of identification	Reference
Kito (ANCF01000106)	WGS	Brazil	*L. interrogans*	N/D	Unknown	2013	NP
LOCaS46 (MT743250 – 66)	gsp genes	Mexico	*L. interrogans*	Canicola	asymptomatic	2020	NP
M10/99 (MG932793)	*secY*	Brazil	*L. interrogans*	Ictero*	Unknown	2018	[46]
M12/20 (OL408068)	*secY*	Brazil	*L. interrogans*	N/D	Unknown	2021	NP
M24/19 (OL408066)	*secY*	Brazil	*L. interrogans*	N/D	Unknown	2021	NP
M60/19 (OL408067)	*secY*	Brazil	*L. interrogans*	N/D	Unknown	2021	NP
M64/06 (MG932794)	*secY*	Brazil	*L. interrogans*	Ictero*	Unknown	2018	[46]
NEG7 (CP093938)	WGS	Brazil	*L. interrogans*	Ictero*	symptomatic	2022	[47]
RCA (MN482693)	16S rRNA	Colombia	*L. interrogans*	N/D	symptomatic	2019	NP
RCA (CP022538)	WGS	Brazil	*L. interrogans*	Ictero*	symptomatic	2015	[48]
SG10 (MW196275)	*sec*Y	Brazil	*L. interrogans*	Ictero*	asymptomatic	2020	[19]
SG13 (MW196276)	*sec*Y	Brazil	*L. interrogans*	Ictero*	asymptomatic	2020	[19]
SG27 (MW196277)	*sec*Y	Brazil	*L. interrogans*	Ictero*	asymptomatic	2020	[19]
SG28 (MW196278)	*sec*Y	Brazil	*L. interrogans*	Ictero*	asymptomatic	2020	[19]
SG29 (MW196279)	*sec*Y	Brazil	*L. interrogans*	Ictero*	asymptomatic	2020	[19]
SG3 (MW196272)	*sec*Y	Brazil	*L. interrogans*	Ictero*	asymptomatic	2020	[19]
SG32 (MW196280)	*sec*Y	Brazil	*L. interrogans*	Ictero*	asymptomatic	2020	[19]
SG33 (MW196281)	*sec*Y	Brazil	*L. interrogans*	Ictero*	asymptomatic	2020	[19]
SG34 (MW196282)	*sec*Y	Brazil	*L. interrogans*	Ictero*	asymptomatic	2020	[19]
SG37 (MW196283)	*sec*Y	Brazil	*L. interrogans*	Ictero*	asymptomatic	2020	[19]
SG54 (MW196284)	*sec*Y	Brazil	*L. interrogans*	Ictero*	asymptomatic	2020	[19]
SG55 (MW196285)	*sec*Y	Brazil	*L. interrogans*	Ictero*	asymptomatic	2020	[19]
SG6 (MW196273)	*sec*Y	Brazil	*L. interrogans*	Ictero*	asymptomatic	2020	[19]
SG69 (MW196286)	*sec*Y	Brazil	*L. interrogans*	Ictero*	asymptomatic	2020	[19]
SG7 (MW196274)	*sec*Y	Brazil	*L. interrogans*	Ictero*	asymptomatic	2020	[19]
SG70 (MW196287)	*sec*Y	Brazil	*L. interrogans*	Ictero*	asymptomatic	2020	[19]
SG73 (MW196288)	*sec*Y	Brazil	*L. interrogans*	Ictero*	asymptomatic	2020	[19]
SG75 (MW196289)	*sec*Y	Brazil	*L. interrogans*	Ictero*	asymptomatic	2020	[19]
SG88 (MW196290)	*sec*Y	Brazil	*L. interrogans*	Ictero*	asymptomatic	2020	[19]
SG95 (MW196292)	*sec*Y	Brazil	*L. noguchii*	N/D	asymptomatic	2020	[19]
SG97 (MW196293)	*sec*Y	Brazil	*L. noguchii*	N/D	asymptomatic	2020	[19]
SG98 (MW196291)	*sec*Y	Brazil	*L. interrogans*	Ictero*	asymptomatic	2020	[19]
UFFB15 (MG932797)	*secY*	Brazil	*L. interrogans*	Ictero*	Unknown	2018	[46]
UFFG001 (MG932795)	*secY*	Brazil	*L. interrogans*	Ictero*	Unknown	2018	[46]
UFFG19 (MG932796)	*secY*	Brazil	*L. interrogans*	Ictero*	Unknown	2018	[46]
USPIA (KR269868)	16S rRNA	Brazil	*L. interrogans*	N/D	Unknown	2015	NP
USPIB (KR269869)	16S rRNA	Brazil	*L. interrogans*	N/D	Unknown	2015	NP
USPIC (KR269870)	16S rRNA	Brazil	*L. interrogans*	N/D	Unknown	2015	NP
USPID (KR269871)	16S rRNA	Brazil	*L. interrogans*	N/D	Unknown	2015	NP
USPIIA (KR269872)	16S rRNA	Brazil	*L. interrogans*	N/D	Unknown	2015	NP

Ictero*: Icterohaemorrhagiae N/D: Not defined; NP: Not published;.
